# Breast cancer risk assessment and risk distribution in 3,491 Slovenian women invited for screening at the age of 50; a population-based cross-sectional study

**DOI:** 10.2478/raon-2023-0039

**Published:** 2023-09-04

**Authors:** Katja Jarm, Vesna Zadnik, Mojca Birk, Milos Vrhovec, Kristijana Hertl, Zan Klanecek, Andrej Studen, Cveto Sval, Mateja Krajc

**Affiliations:** Sector for Cancer Screening and Clinical Genetics, Institute of Oncology Ljubljana, Ljubljana, Slovenia; Faculty of Medicine, University of Ljubljana, Ljubljana, Slovenia; Faculty of Health Sciences, University of Primorska, Izola, Slovenia; Sector for Oncology Epidemiology and Cancer Registry, Institute of Oncology Ljubljana, Ljubljana, Slovenia; Faculty of Mathematics and Physics, University of Ljubljana, Ljubljana, Slovenia

**Keywords:** breast cancer screening, personalised screening, risk assessment, IBIS, Tyrer-Cuzick model, breast density

## Abstract

**Background:**

The evidence shows that risk-based strategy could be implemented to avoid unnecessary harm in mammography screening for breast cancer (BC) using age-only criterium. Our study aimed at identifying the uptake of Slovenian women to the BC risk assessment invitation and assessing the number of screening mammographies in case of risk-based screening.

**Patients and methods:**

A cross-sectional population-based study enrolled 11,898 women at the age of 50, invited to BC screening. The data on BC risk factors, including breast density from the first 3,491 study responders was collected and BC risk was assessed using the Tyrer-Cuzick algorithm (version 8) to classify women into risk groups (low, population, moderately increased, and high risk group). The number of screening mammographies according to risk stratification was simulated.

**Results:**

57% (6,785) of women returned BC risk questionnaires. When stratifying 3,491 women into risk groups, 34.0% were assessed with low, 62.2% with population, 3.4% with moderately increased, and 0.4% with high 10-year BC risk. In the case of potential personalised screening, the number of screening mammographies would drop by 38.6% compared to the current screening policy.

**Conclusions:**

The study uptake showed the feasibility of risk assessment when inviting women to regular BC screening. 3.8% of Slovenian women were recognised with higher than population 10-year BC risk. According to Slovenian BC guidelines they may be screened more often. Overall, personalised screening would decrease the number of screening mammographies in Slovenia. This information is to be considered when planning the pilot and assessing the feasibility of implementing population risk-based screening.

## Introduction

Breast cancer (BC) is the most common cancer in women in developed countries. Globally, more than 2,200,000 women were diagnosed with BC in 2020 – 355,457 only in the European Union.^1,2^ The average crude incidence rate in Slovenia has risen from 32.3/100,000 between 1965 and 1969 to 139.8/100,000 women in the period from 2015 to 2019. Between 2015 and 2019, the average annual number of new BC cases in Slovenia was 1,454 (the female population in 2019 was equal to 1,044,783).^3,4^ According to estimated age-standardized incidence rate (European standard) in 2020, Slovenia ranks 18^th^ among EU member countries.^2^

Breast cancer burden is increasing mainly due to an ageing population. Moreover, many other risk factors affect BC predisposition. The most important are reproductive risk factors (early menarche, later age at first full-term pregnancy, nulliparity and late menopause affecting the levels of endogenous hormones), hormone use (intake of exogenous hormones, hormone replacement therapy), some lifestyle factors (alcohol use, overweight and physical inactivity), a high mammographic breast density, benign breast diseases (proliferative disease without atypia and atypical hyperplasia), anthropometric characteristics (height, weight) and genetic susceptibility.^5,6^

In addition to primary prevention, secondary prevention of BC with screening can be very successful in reducing BC mortality rates in organised population–based cancer screening programmes and with an uptake over 70%.^7^ BC screening programmes in the European Union member states offer standard screening for all women aged 50–69 (in certain cases 40–74) based on a single risk factor, age as an entry criterion.^8^ The latest European Commission recommendations from December 2022 recommend mammography screening in women aged 50–69 and suggest screening in wider age intervals, 45–74 if feasible.^9^ The evidence shows that there is high certainty that mammography screening reduces the risk of BC mortality in women aged 50–69 (138 to 483 deaths averted per 100,000 women screened). In addition, women invited to screening show a lower risk of BC being diagnosed in advanced stages, regardless of age group. However, there is also moderate certainty for undesirable effects of screening, e.g. overdiagnosis and false-positive results associated with an increased number of invasive procedures and women's distress.^10^

As already mentioned, the age is not the only risk factor and other risk factors can also contribute to the development of BC. Therefore, the one-size-fits-all approach does not take into account the heterogeneity of the BC biological subtypes nor the different BC risks in the population.^6^ New scientific data suggest that a new screening strategy based on the estimation of individual BC risk may have a better harms/benefits ratio for women in comparison to the current standard age-based screening. A personalized approach can tailor screening strategies according to women's risk. In fact, the Guidelines development group of experts at European Commission Initiative on Breast Cancer (ECIBC) supports the priorities in the field of mammography screening that include identification of risk factors for stratifying women into different risk groups; to find those who should start with the screening earlier and might be screened with shorter intervals.^8^ Some studies have already been conducted and some randomized controlled trials are ongoing. These studies want to test the hypothesis that an age-based BC screening strategy, where the screening policy is the same for all women in the target population, is not optimal and risk-based screening over current one-size-fits-all screening strategy should be recommended to improve the harms/benefits ratio.^11^

Various mathematical models for calculating individual BC risk are known today, to name just a few of them: the Gail model, the Breast Cancer Surveillance Consortium (BCSC) risk calculator, the Tyrer–Cuzick model, The Breast and Ovarian Analysis of Disease Incidence and Carrier Estimation Algorithm (BOADICEA) and an online tool enabling healthcare professionals to calculate an individual's future risks of developing breast and ovarian cancer using cancer family history, genetic and other risk factors (CanRisk model).^12,13,14,15,16^

In Slovenia, more than 100,000 screening mammographies are performed every year in the target population, inviting approx. 280,000 BC-free women aged 50 to 69 to the Slovenian BC screening programme every two years.^17^ In addition, women at high and moderately increased risk are currently identified and assessed at the Institute of Oncology Ljubljana at the Department of Clinical Cancer Genetics. Cancer genetic counselling, genetic testing, personalised cancer screening and risk reduction strategies are offered when a woman's BC risk is more than doubled in comparison to the general population's BC risk. Since 1999, women have been selected due to positive family history, and genetic testing is offered when indicated according to the Slovenian BC diagnostic and treatment guidelines.^18^ Breast cancer risk is currently assessed either by using the Tyrer-Cuzick or the CanRisk tool and personalised surveillance is offered to women at higher risk. For the general population with a lifetime risk under 15% (population risk), Slovenian guidelines recommend regular breast self-examination, early recognition of BC symptoms and signs, and participation in the Slovenian BC screening programme. For women with moderately increased BC risk, additional yearly clinical breast examination and yearly mammography are recommended. Risk should be identified using mathematical models, e.g. Slovenian International Breast Cancer Intervention Study (S-IBIS) evaluation tool or cancer risk (CanRisk) tool based on reliable family history, which should be verified whenever possible in the cancer registry.^18,19^

No population-based cross-sectional study for assessing the BC risk in the Slovenian population invited for BC screening has been performed yet.

The aims of the study were (i) to assess the feasibility of BC risk assessment in Slovenian women when invited to the BC screening programme, (ii) to identify the distribution of women in the BC risk groups (low, population, moderately increased, and high risk) through assessing the 10-year and lifetime BC risk, by using also the information on the breast density that is not yet routinely available as a part of the standardized mammography report and (iii) to assess the number of screening mammographies in case risk-based screening would be implemented according to different screening protocols.

## Patients and methods

Our study was a cross-sectional population-based study and enrolled 11,898 women at the age of 50 invited to BC screening in 2021 (birth cohort 1971). The National Medical Ethics Committee at the Ministry of Health of the Republic of Slovenia (No. 0120-244/2018/4) approved the study. The first 3,491 questionnaires (out of 6,785 returned) were analysed for the purpose of this article. Entering all received questionnaires into the database and arranging the data was a lengthy process, so the first half of refined data was analysed preliminarily, since the sample size was already adequate (minimum sample size would be 800).

### Participants recruitment and materials

Women turning 50 years are invited with a personal letter to the Slovenian BC screening programme to participate in mammography screening organized according to the EU guidelines.^17,20^ All eligible women aged 50 (with no previous BC diagnosis) in 2021, were sent a self-administrated structured 5-page questionnaire via postal mail together with a screening invitation and explanatory text to sign informed consent for participating in this study. The family history questionnaire was adopted from the one used at the Department of Clinical Cancer Genetics, encompassing questions about anthropometric, reproductive and hormonal anamnesis and family history (Table 1).^21^ In addition, just for the purpose of this study, breast density was assessed by the radiologist using the mediolateral oblique view of the screening mammograms, in accordance with the BI-RADS 5^th^ edition reporting system that classifies breast density into four levels.^22^

**TABLE 1.** Questionnaire content used for Slovenian International Breast Cancer Intervention Study (S-IBIS) evaluation tool score calculation

Factors affecting levels of endogenous hormones (menarche, menopause, first birth age).
Exogenous hormone intake (menopause hormone replacement therapy).
Anthropometric characteristics (height, weight).
Breast biopsy (done, not done, presence of atypia, atypical hyperplasia).
Family history of breast and ovarium cancer (mother, sisters, half-sisters, daughters, grandmothers, aunts, male relatives).
Ovarian cancer of the participants.

For menopausal status, the time interval between the date of filling in the questionnaire and the date of women's last menstrual period was considered, 31 and 365 days being cut-offs for the groups (premenopausal, perimenopausal and postmenopausal).^23^ For participants’ description, the characteristics of study participants were grouped into categories according to the relative risk caused by the risk factors incorporated in the Tyrer-Cuzick model.^14,24^ The denominator for calculating the percentage of frequencies was the sum of participants (3,491).

### Risk calculation

In the Slovenian national health system, Slovenian IBIS is ready for use allowing an evidence-based assignment of an asymptomatic individual to a group of population, moderately increased and high BC risk. The S-IBIS software was developed at the Institute of Oncology Ljubljana in 2018 within a research project.^19^ It is an adjustment of the IBIS software with the Tyrer-Cuzick algorithm, where Slovenian generation-specific population BC risks were applied and it is specifically designed to calculate the individual risk of BC in Slovenian women. BC incidence and mortality rates between 2006 and 2010 were obtained from the population-based Slovenian Cancer Registry.^25^ The Tyrer-Cuzick algorithm is recognized as one of the most consistent models and validated on several populations. It calculates both, a 10-year risk and a lifetime risk of BC based on the women's personal, reproductive, and family characteristics.^14^ Moreover, the latest version of the model (version 8) incorporates mammographic density.^26^

After calculating the risk for each individual (first with breast density included and then without breast density), study participants were grouped into risk categories according to relative risk that was calculated with the Tyrer-Cuzick model.^14,24^ The cut-offs for the distribution of individuals into the low, population, moderately increased and high risk categories for BC are shown in Table 2. Lifetime risk is defined as the risk of developing BC by the age of 85.^19,25^ In case of missing or unknown data, the population reference relative risk was considered.

**TABLE 2.** Breast cancer risk categories for Slovenian women at the age of 50 and risk-based screening scenarios according to different protocols^18,24,26^

**Breast cancer risk category**	**Lifetime risk**	**10-year risk**	**Slovenian risk-based screening guidelines^18,25^**	**PROCAS study protocol^27^**
Low risk (%)	^**^	< 1.3	^**^	5-year mammography screening interval
Population risk (%)	< 16	1.3–3.9	2-year mammography screening interval	3-year mammography screening interval
Moderately increased risk (%)	16–30	4.0–6.5	1-year mammography screening interval	2-year mammography screening interval
High risk (%)	> 30	> 6.5	1-year mammography screening interval	1-year mammography screening interval

** not applicable; PROCAS = Predicting Risk of Breast Cancer at Screening

The number of screening mammographies was estimated for the same group of women (N = 3,491, considering all would attend the screening regularly by the age of 69) for two different risk-based scenarios/protocols according to the Slovenian BC diagnostic and treatment guidelines and English Predicting Risk of Breast Cancer at Screening (PROCAS) cohort study (Table 2).^18,25,27^

The tool SPSS, version 24 (IBM Corp., Armonk, NY, USA), R Project for Statistical Computing (v4.3.2) and RStudio (2023.03.0, R Core Team 2023) were used for the statistical analyses and risk calculations. Statistical significance was determined using the Clopper-Pearson 95% confidence intervals.

## Results

In total, 57% (6,785/11,898) of women who received the Slovenian BC screening programme invitation consented to the study and filled in the questionnaire. The first 3,491 returned questionnaires were included in the BC risk assessment and analysed. The characteristics of the study participants are listed in Table 3.

**FIGURE 1.**
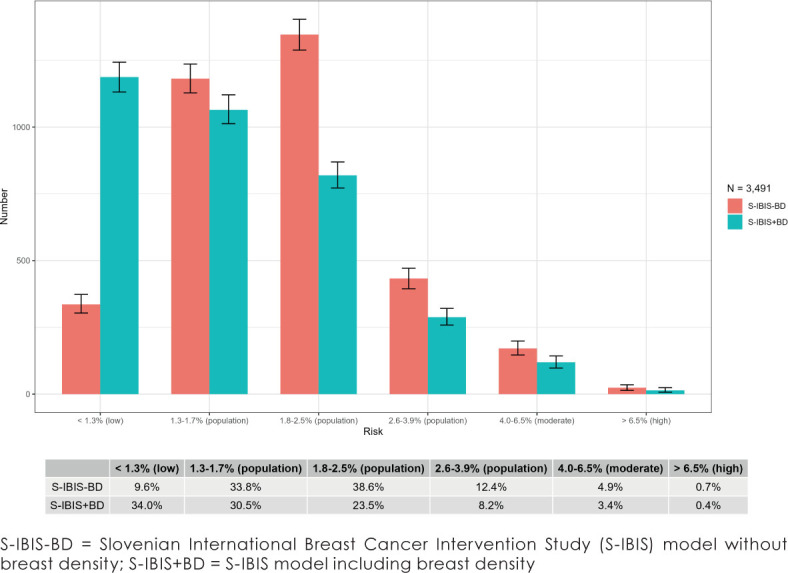
10-year breast cancer risk distribution in Slovenian women, aged 50 years, by using S-IBIS without breast density data and with breast density data. Clopper-Pearson 95% confidence intervals are shown.

**TABLE 3.** Characteristics of study participants (n = 3,491)

	**Frequency (N)**	**Per cent (%)**
Family history: first-degree relatives with breast/ovarian cancer (mother, father, sisters, daughters)		
positive (1 relative)	326	9.3
positive (2 or more relatives)	28	0.8
negative	2,854	81.8
unknown	283	8.1
Family history: second-degree relatives with breast/ovarian cancer (aunts, uncles, grandmothers, half-sisters)		
positive (1 relative)	437	12.5
positive (2 or more relatives)	148	4.2
negative	2,552	73.1
unknown	354	10.1
Age (years) at menarche		
< 13	1,138	32.6
13	940	26.9
> 13	1,339	38.4
unknown	74	2.1
Age (years) at first birth		
< 25	1,570	45.0
25–28	736	21.1
29–34	659	18.9
35	191	5.5
nulliparous	335	9.6
unknown	0	0.0
Menopausal status		
premenopausal	1,392	39.9
perimenopausal	632	18.1
postmenopausal	631	18.1
unknown	836	23.9
HRT usage		
yes	286	8.2
no	3,123	89.5
unknown	82	2.3
Breast biopsy		
no biopsy	3,123	89.5
biopsy (hyperplasia, atypical hyperplasia, LCIS)	21	0.6
biopsy (else)	44	1.3
biopsy (unknown result)	256	7.3
unknown if biopsy done	47	1.3
Breast density		
BI-RADS a	215	6.2
BI-RADS b	1,802	51.6
BI-RADS c	1,410	40.4
BI-RADS d	34	1.0
unknown (no screening mammography)	30	0.9
BMI		
< 19	48	1.4
19–25	1,165	33.4
> 25	1,336	38.3
unknown	942	27.0

BI-RADS = breast imaging-reporting and data system^22^; BMI = body mass index; HRT = hormone replacement therapy in menopause; LCIS = lobular carcinoma in situ

The majority of women were parous (90.4%) and did not have any first (89.9%) or second-degree (83.2%) relatives affected. Further, 39.9% of women included in the study were premenopausal, 18.1% perimenopausal and 18.1% postmenopausal, 23.9% of them did not report the date of their last period. Eight-point two percent of women reported being current or previous users (in the last 5 years) of hormone replacement therapy (HRT). More than a half (51.6%) of women were assessed with BI-RADS b score for breast density, and 40.4% with BI-RADS c score.^22^ More than two-thirds of women reported their data for the risk factors, e.g. the age at menarche, the age at first childbirth, menopausal status, HRT use and breast biopsy. Moreover, breast density was assessed for 99% of participants.

### Frequencies of BC risk

Table 4 shows the frequencies of BC risk in the study population; 3.4% of participating women were assessed with moderately increased 10-year BC risk, and 2.2% with moderately increased lifetime risk. Only a small proportion of women had high 10-year and lifetime risk (0.4% and 0.03%, respectively). The mean of the 10-year risk was found to be 1.7% with a standard deviation of 1.0, and the mean of the lifetime risk was 6.3% with a standard deviation of 3.2. Among 3,491 analysed study participants, 27 were diagnosed with breast cancer after first screening mammography in 2021; one of them was assessed as high risk (10 years BC risk), none as moderately increased, nine as low risk and 17 as population risk.

**TABLE 4.** 10-year and lifetime breast cancer risk frequencies in the study group (risk calculated with the Slovenian International Breast Cancer Intervention Study (S-IBIS) evaluation tool, breast density information included) (N = 3,491)

**Risk category**	**Frequency (N)**	**Per cent (%)**
10-year breast cancer risk		
low	1,186	34.0
population	2,172	62.2
moderately increased	119	3.4
high	14	0.4
Lifetime breast cancer risk		
population	3,413	97.8
moderately increased	77	2.2
high	1	0.03

### Breast density information

Adding breast density information to the Tyrer-Cuzick model (10-year BC risk) significantly changed the distribution of women in our study. This information moved the majority of women to lower-risk groups. The proportion of women in high, moderate and upper-population risk groups (2.6%–3.9%) was also decreased, shifting women to lower–population (1.3%–1.7%) and low risk (below 1.3%) groups (Figure 1).

### Number of screening mammographies when different risk-based screening protocols are applied

Table 5 shows the change of the number of screening mammographies in the screening programme when considering different screening protocols (age-based and risk-based using the S-IBIS model including breast density [S-IBIS+BD]) applying two different risk-based protocols, described in the Slovenian guidelines and in the PROCAS study.^18,27^

**TABLE 5.** Number of screening mammographies among 3,491 women through their whole screening period (aged 50–69 years) in case of different screening scenarios

**Risk category**	**Lifetime breast cancer risk**		**10-year breast cancer risk**	

	**Age-based screening (current screening)^17^**	**Slovenian risk-based screening^18^**	**Age-based screening (current screening)^17^**	**PROCAS risk-based screening^27^**
Low risk	–	–	11,860	4,744
Population risk	34,130	34,130	21,720	15,204
Moderately increased risk	770	1,540	1,190	1,190
High risk	10	20	140	280
Total	34,910	35,690	34,910	21,418

PROCAS = Predicting Risk of Breast Cancer at Screening

When considering applying the current Slovenian risk-based screening guidelines for lifetime BC risk, 2.2% (|35,690–34,910|/34,910) more mammographies compared to the current screening strategy would be performed in 20-year screening period (aged 50 to 69 years).

Considering the PROCAS protocol for 10-year BC risk, the number of screening mammographies in the risk-based screening would drop by 38.6% (|21,418–34,910|/34,910) in the 20 years compared to the current screening. It considers enhancing the screening intervals among above-average BC-risk women, but also less frequent screening intervals in the below-average risk group. An increase of 100.0% (|280–140|/140) in the number of mammographies would be observed in the high risk group and a decrease of 40.6% (|19,948–33,580|/33,580) in the low and population risk groups. In fact, even in case of risk stratification using S-IBIS with no breast density information less mammographies would be performed considering PROCAS protocols for 10-year BC risk, namely 30.5% (|24,254–34,910|/34,910) less.

## Discussion

This cross-sectional population-based study enrolled 11,898 women at the age of 50 who had no previous BC diagnosis and were invited to the Slovenian BC screening programme in 2021. The first 3,491 questionnaires (out of 6,785 returned) were analysed. For each woman, risk factors data was collected and BC risk was calculated using the S-IBIS calculator. Women were classified into BC risk groups (low, population, moderately increased and high) according to Slovenian specific population-based cut-offs for distribution into risk groups (Table 2).^25^

To sum up the study objectives, (i) study up-take was satisfactory: 57% of women invited to the study returned BC risk questionnaires, proving the feasibility of BC risk assessment along with the screening participation; (ii) after individual risk calculation with breast density information included, 34.0% of responders were assessed with low, 62.2% with population, 3.4% with moderately increased and 0.4% with high 10-year BC risk and 97.8%, 2.2% and 0.03% with population, moderately increased and high lifetime risk, respectively; and finally, (iii) the number of screening mammographies would decrease for more than one third in case of PROCAS study risk-based screening protocol, as it was described in the previous and current European studies.^27,28^

### Study uptake

In our study, we experienced a satisfactory up-take (57%). Our results confirm the feasibility of determining BC risk at the entry in the Slovenian BC screening programme and this result is in accordance with literature reports. The 1971 birth cohort of women eligible for screening was reached and women were offered preventive mammography screening and voluntary study participation. Therefore, no additional interventions were anticipated. The invitation to the study was part of a regular screening programme. Furthermore, the women recognized as high risk for BC will be offered genetic counselling, where BC risk factors will be reverified following the clinical pathway and BC risk will be recalculated by using verified data and genetic data where applicable.^21^ As reported in the PROCAS study, the majority of women (95%) indicated they wished to receive risk information.^29^ Also, the DECIDO study showed a positive attitude and a high understanding of risk-based screening.^30^

### Risk stratification and comparison with other studies

According to our results, 3.4% of Slovenian women invited for mammographic screening at the age of 50 and consented to participate in our study belong to the moderately increased 10-year BC risk group and 0.4% to the high risk group and would have to be screened more often according to our guidelines. So far, these data were unavailable, since no population-based cross-sectional study has been performed to assess the BC risk in Slovenian women. Some regional and hospital/breast centres-based research have been conducted to assess women's BC risk.^19,25,31,32^ Due to different subpopulations assessed and low sample volumes, the results of these studies cannot be directly compared to our study. They were all conducted among women referred to breast centres, where women with a positive family history or previous biopsies are normally assessed. Therefore, we may predict their BC risk could be higher when compared to the general population.

In 2016, a prospective cohort study among 100 asymptomatic women (aged 20–49) from one regional breast centre was conducted, testing the S-IBIS calculator (version 8) for lifetime BC risk. 18% of women were identified as moderately increased risk and none at high risk (above 30% risk). 86% of women referred for another mammography in 12 months would not need annual screening mammography. This study proved the S-IBIS is effective in decreasing the number of referrals for annual mammography.^31^ In 2018, a study recruiting women from regional breast units proved that 148 (75.1%) out of 197 interviewed and examined women were assigned to the population risk category, 49 (24.9%) to the moderately increased risk category and none to the high or low risk categories.^25^ For that study, the S-IBIS tool (version 8) was used and tested.^18^ Another Slovenian study from 2020 was assessing the proportion of women with above average 10-year risk of BC (more than 2%) using the S-IBIS calculator version 8 (breast density not considered). All assessed women in the study were already at higher BC risk at the baseline. They were either healthy with some breast symptoms or already diagnosed with BC (for the latter, the data prior to BC diagnosis was considered); 48.7% and 39.2% of women were recognised with above average BC risk, respectively. The study concluded, that inclusion of additional risk factors into the S-IBIS is needed to reliably stratify women into the BC risk groups.^32^

As shown above, the advantage of BC risk assessment is the use of S-IBIS, a BC risk calculator in the Slovenian breast centres, which could reduce the number of unnecessary preventive mammographies for the majority of women assessed with the population risk, giving room for symptomatic women and women at moderately increased and high risk. This was proved reasonable in all aforementioned Slovenian risk studies.^25,31,32^

Furthermore, risk-based screening is already ongoing in several countries across the world, but at the moment only in study settings. The English cohort study PROCAS conducted in the UK in the period from 2009 to 2020 and recruiting 63,000 women in two large studies (aged 50–70), concluded that the Tyrer–Cuzick risk prediction model (version 6) accurately predicts BC risk.^27^ However, some further improvements are still required. The study showed that 11% of women in the general population have moderately increased BC risk and 85% of women have average population risk or very low risk. It also indicated that adding breast density and genetic information improved risk precision and can be used to tailor screening. Using a combination of both predicts that 70% of the population with average or below-average risks have very low rates of advanced BC. Moreover, 3-yearly screening interval appeared effective in 70% of the population in the UK. Additionally, giving women their risk information and management feedback increased their next screening participation, and even more, it encouraged them to improve their lifestyles.^27,33^ In numbers, 24% of participating women were found at low 10-year risk, 61% at average (population) risk, 11% at moderate and 4% at high with breast density information added to the Tyrer-Cuzick model (version 6).^27,33^ Similar proportions were found in our study – 34%, 62%, 3%, and less than 1%, respectively. The difference, however, may be due to the age gap – only 50-year-olds in our study vs. women aged 50–70 in the PROCAS study. Furthermore, if we transpose the risk groups’ distribution from our study to the PROCAS potential risk-based protocol (Table 2 and Table 5), the number of screening mammographies will decrease by more than one-third (38.6%) in 20 years of screening compared to current screening programme workload. The main reason is the longer screening interval for the majority of women with population BC risk.

At the moment, two big randomized controlled trials (RCT) are trying to answer whether personalized screening is non-inferior to the standard age-based screening protocols. WISDOM is a multicentre RCT ongoing in the USA, comparing risk-based screening to annual screening in women aged 40–74 years and determining whether risk-based screening is as safe as annual mammographic screening, which is the screening policy in that country.^34^ Similarly, an European RCT ongoing in 6 countries is called MyPeBS (My Personalized Breast Screening).^28,35^ Study MyPeBS is an international randomized, multicentric study assessing the effectiveness of a risk-based BC screening strategy compared to a standard screening in detecting stage 2 or higher breast cancers. It will recruit 85,000 women from Belgium, France, Israel, Italy, the United Kingdom and Spain. Each participating country has different current national guidelines – biennial or triennial mammography screening beginning from the age of 40 to 50 years and ending from 69 to 74 years.^28,35^ Both RCTs are integrating polygenic risk scores (with 313 single-nucleotide polymorphisms) in the risk calculations, for which BCSC and Tyrer-Cuzick calculators are used.^34,35^ Risk score 313 provides the highest level of BC risk stratification in the population, followed by mammographic breast density and other risk factors.^6^ Those RCTs are aimed at investigating whether the personalised approach is at least equally or more appropriate than the standard one.^35^

Our results also show that the vast majority of women (96.2%) have low (34.0%) or population (62.2) 10-year BC risk and are thus appropriately screened every 2 years, according to Slovenian and NICE guidelines.^18,36^ However, 3.8% of women at the age of 50 would need more intensive BC surveillance.

### Data accuracy

Regarding the reliability of the data collected, risk feedback in PROCAS (in person or by telephone) showed that women's information on their risk factors stated in the questionnaires was not always accurate, and in some cases, women changed risk groups after consultation. The greatest proportion of changes in risk occurred in those originally assessed as having a 10-year TC risk of ≥ 8%.^27^ With this in mind, the proportion of the low and population risk groups in our study may be overestimated (listed in study limitations), since the self-administrated questionnaire was quite long and demanding for an average user. Besides, it may have deterred some women from participating and entering the complete information about risk factors. Some women may have not enquired about the cancer history of their family members, especially distant relatives. However, we expect, that positive cancer diagnoses are well-known in families and that women with the highest risk were not missed.^37^ Overall, risk scores should be calculated with verified data on risk factors, where more effort to obtain accurate data should be considered. The risk feedback to women (and consultation, if possible) is an example of how to improve the data accuracy for risk identification, as it is planned for our identified high risk women.

The analysed study women are representative group of the Slovenian population. In Slovenia, 10% of women at the age of 50 are nulliparous (in our study 9.6%) and 50% of women at the age of 45 to 54 are overweight or obese (body mass index higher than 25) – in our study 38.3%.4,38 Noteworthy, 27% of study participants did not provide the data on body weight or height.

### Breast density

In addition to the data collection, mammographic density is a strong independent risk factor for BC and it is not a part of standard screening mammography results.^39^ For almost all women participating in our study, breast density was estimated and the majority were assessed with breast density BI-RADS b (51.6%) and BI-RADS c (40.4%). These results are in accordance with the literature reports.^40,41^ However, it is known that the BI-RADS assessment method is subjective and depends on the reader, reading volume and image quality.^42^ In the PROCAS study, the percentage of women in each risk category changed when density was added to the Tyrer-Cuzick risk model. Adding density moved many women from average (population) to higher or lower risk of developing BC.^27,33,43^ On the contrary, in our study, adding density mainly moved women from higher-risk groups to lower-risk groups (shift to the left) (Figure 1). We can assume that density contributes to the model to decrease the risk at the age of 50 (in the PROCAS study, women's age was 50–70, the majority of women were assessed for breast density as BIRADS b (lower density)).

### Potential risk-based screening strategies

With risk-based screening and following the Slovenian BC guidelines, the number of mammographies increased (by 2.2%) on account of women with above population risk. Less frequent screening in women with lower BC risk is not considered at this point.^18^ It is clear that communicating the reduction in screening frequency in the general population is rather demanding even though more screening does not prove higher efficiency.^28,44^ In reality, this is the biggest uncertainty in the risk-based screening, because it is very unlikely that less screening would be accepted in the target population. While recruiting participants for the MyPeBS study, 60% of women recognised as low risk, opted-out the intervention group due to prolonged screening intervals.^45^ However, this ongoing RCT in Europe does predict less frequent screening intervals for women with population and low risk, i.e. 4 years.^28^ Therefore, we can expect a smaller amount of annually performed screening mammographies in the national BC screening programme in case risk-based screening protocol that includes less frequent screening for lower-risk women is recommended. Nevertheless, at the time there is not enough evidence for such recommendation at the Europe level, since not many prospective RCTs are being conducted nor concluded.

For Slovenian situation as for the other countries, first, communicating clinical safety of less intensive screening can be an important obstacle, and secondly, the risk-based screening protocol should not be to complex to be feasible and to be able to follow-up the participants efficiently. To add, recalculation of 10-year BC risk should be considered after 10 years.

### Study strengths

For the first time in Slovenia, a population-based sample of women was assessed for BC risk and it is the first time potential changes in the organised screening programme in case of introducing risk stratification have been estimated.

The mammographic density was assessed exclusively for our study (available for 99% of our participants), which made it possible to define BC risk more accurately and proved to be feasible to incorporate this information into screening data. Equally important, the IBIS software has recently been adjusted using Slovenian-specific population BC risks making it more valid.^19^ Women's uptake to study participation (57%) was satisfactory when compared to other studies. In the same year, the screening participation rate of women aged 50 was 74.4%.^46^ In the PROCAS study (first phase of recruitment similar to ours, where all women invited for BC screening were sent a participant invitation letter), screening uptake was 68% and study uptake 37%.^27^

### Study limitations

The study questionnaires were self-administrated and data verification by enquiring with the women in person or using the health records was not performed. Furthermore, the family members’ names were not collected so the family cancer history was not medically confirmed; some women did not remember all the family cancer diagnoses. In addition, some may wrongly interpret the topography of cancer (e. g. ovarium cancer instead of cervical cancer). Additionally, the health literacy of women is very variable, which can affect answering the questionnaire without explanations. Thus, some data we used might be unreliable, and probably we have under or overestimated the risk scores. To improve data quality, assistance with filling out the risk questionnaires is needed. From clinical work it is known, that majority of people cannot finish the risk tool/questionnaire without assistance. In practice for risk-based population-wide screening this means, that trained radiographers or administrative personnel should administer women at their first screening visit to gather adequate information. Besides, legal framework for data verification through other databases (like cancer registry and registry of genetically tested individuals) should be legislated.

We assume that some women did not participate in the screening programme nor in this study since they have already been regularly and thoroughly surveilled at the Institute of Oncology Ljubljana in the High risk breast clinic. After the BC risk had been assessed in the Department of Clinical Cancer Genetics, which has been operating for more than 20 years, women with above-population BC risk were referred to the High risk breast clinic at the Institute of Oncology Ljubljana.^21^ For this reason, a certain number of women with moderately increased and high risk for BC may not have been considered in our study (selection bias), thus decreasing the proportion of women in higher-risk groups.

In conclusion, assessing a personalised risk score at a woman's first screening appointment is reasonable. It can improve screening benefits for low and higher-risk groups in the target population. To plan more efficiently the BC screening and BC patients’ care if the risk-based screening over the current one-size-fits-all screening strategy would be evidence-based and recommended in the future, we assessed the BC risk in a 50-year old cohort of women in Slovenia and found the majority of women belonging to the population 10-year BC risk (62.2%) and 3.4% to moderately increased BC risk group. Our evidence supports the effectiveness of the current Slovenian screening protocol for the majority of screened women. Potential future risk-based screening would change the manner of BC screening for approximately one third of Slovenian women (38% at high, moderately increased, or very low risk) with either additional screening methods at a higher frequency or with prolonged screening intervals, respectively. Risk assessment is feasible at the entry to screening. Due to the study uptake, where more than half of screened women took part, rather high risk assessment uptake among Slovenian women is expected. However, data accuracy can be improved with in-person risk assessment and risk counselling.

Still, only randomised and observational studies will answer the main question regarding personalised BC screening in the future. And this is if risk-based screening over the current one-size-fits-all strategy should be recommended.
